# Identification of *BmSP25* gene in *Bombyx mori* with antiviral function against *BmNPV*

**DOI:** 10.1371/journal.pone.0345502

**Published:** 2026-03-27

**Authors:** Yonghong Zhang, Yajin Li, Zhengqin Wang, Jiafu Luo, Lingli Li, Hongrui Zhang

**Affiliations:** 1 College of Plant Protection, Yunnan Agricultural University, Kunming, Yunnan Province, China; 2 Sericulture and Apiculture Research Institute, Yunnan Academy of Agricultural Science, Mengzi, Yunnan Province, China; Guangzhou University, CHINA

## Abstract

*Bombyx mori* serine protease (BmSP) is constitute a gene family of proteolytic enzymes characterized by serine residues at their active sites and play critical roles in physiological processes, including digestion, growth and development, and immune responses. As a member of the BmSP family, BmSP25 exhibits differential expression following *Bombyx mori* nucleopolyhedrovirus (*BmNPV*) infection. Our result demonstrated that *BmNPV* infection upregulated BmSP25 expression in both resistant (SuN) and susceptible (P50) silkworm strains, with a more pronounced response observed in SuN than in P50. To further investigate its function, siRNA-mediated knockdown of *BmSP25* in BmN cells promoted the proliferation of recombinant BV-EGFP virus, whereas overexpression of *BmSP25* significantly suppressed *BmNPV* replication. To validate its antiviral activity at the organismal level, transgenic silkworm strains overexpressing *BmSP25* (*BmSP25*-OE) and BmSP25 knockout strains (*BmSP25*-KO) were generated. Following oral inoculation with *BmNPV*, viral proliferation was significantly inhibited in the *BmSP25*-OE strain, whereas viral replication was notably enhanced in the *BmSP25*-KO strain. This study is the first to clearly demonstrate the anti-*BmNPV* function of BmSP25 in silkworms, providing a foundation for further elucidation of its role in host immune defense mechanisms and identifying a potential genetic target for molecular breeding aimed at improving disease resistance in silkworms.

## Introduction

The silkworm, *Bombyx mori* L. is an economically important insect and a representative Lepidopteran model organism [1,2]. During sericulture, silkworms are highly susceptible to infection by multiple viruses, among which with double-stranded DNA (dsDNA) and double-stranded RNA (dsRNA) viruses pose particularly serious threats. *Bombyx mori* cytoplasmic polyhedrosis virus (*BmCPV*), a dsRNA virus, infects midgut epithelial cells of silkworm and subsequently induces host innate immune response, including upregulation of the *BmToll-2* gene [3]. Among dsDNA viruses, *BmNPV* also triggers immune responses in the midgut following oral infection. Silkworms are frequently infected with *BmNPV* during sericulture, leading to blood-type grasserie and severe losses in silk production. As a major viral pathogen in sericulture, yet effective control measures remain lacking, posing a substantial challenge to the industry. *BmNPV* belongs to the family Baculoviridae, featuring and is characterized by rod-shaped enveloped virions containing a closed circular double-stranded DNA genome of approximately 130 kb [4,5]. Viral occlusion bodies (OBs) enter the midgut lumen of silkworms through ingestion. Under alkaline digestive conditions, OBs dissolve and release virions, which subsequently binding to columnar epithelial cells of the midgut and spread to other healthy tissues [6]. The silkworm midgut therefore functions as the first barrier against *BmNPV* infection and represents a critical site for virus host interactions. Previous studies have identified several midgut-expressed proteins exhibiting with anti-*BmNPV* activity [7–12]. Consequently, elucidating the roles of genes highly expressed genes in midgut during *BmNPV* infection is essential crucial for understanding the immune defense mechanisms of silkworms.

Different silkworm strains display distinct levels of resistance to *BmNPV* infection. For example, KN [13], NB [14], and A35 [15] are highly resistant strains, whereas 306 and P50 are susceptible. With advances in silkworm molecular biology, the functions of several genes and proteins associated with *BmNPV* resistance have been characterized elucidated. For instance, knockout of the peptidoglycan recognition protein 2 (BmPGRP2−2) gene suppresses *BmNPV* proliferation in both cell lines and silkworm larvae, thereby reducing host mortality [16]. In contrast, overexpression of BmAtlastin-n [17], silkworm lysozyme (BMC-LZM) [18], and silkworm alkaline trypsin A (BmTA) [19] in larvae or cells enhances resistance to *BmNPV*. Additionally, silkworm serine/threonine protein phosphatase 2A (BmPP2A) has been shown to possess anti-*BmNPV* activity [20]. Conversely, proteins such as receptor expression-enhancing protein a (BmREEPa), which interacts with GP64 [21], the E3 ubiquitin-protein ligase SINAL10 which binds to GP64 [22], and the autophagy-related protein ATG13 [23] have been demonstrated promote *BmNPV* proliferation in BmN cells.

Serine proteases are widely distributed among insects and play essential roles in intestinal protein digestion. The midgut of Lepidopteran larvae contains a complex repertoire of proteolytic enzymes with diverse substrate distinct specificities, including trypsin, chymotrypsin, elastase, cathepsin-B-like proteases, aminopeptidases, and carboxypeptidases, which collectively participate in protein digestion [24]. In the larval intestinal environment, serine proteases predominate, accounting for approximately 95% of total digestive enzyme activity [25]. Serine proteases are endopeptidases characterized by a serine residue at the active site that functions as a nucleophile. These enzymes exhibit diverse biological functions, being directly involved in critical physiological processes such as protein metabolism, digestion, blood coagulation, apoptosis, immune regulation, development, and fertilization [26,27], while regulating multiple synergistic processes through proteolysis. Zhao *et al*. [28] identified 143 serine protease-related genes in the silkworm genome database based on amino acid sequence homology with serine proteases from other species, designated BmSP1 to BmSP143, including 51 serine protease (BmSPs) and 92 serine protease homologs (BmSPHs). Members of the BmSP family exhibit functional diversity, with distinct expression patterns across different tissues and developmental stages of the silkworm, thereby fulfilling a wide range performing diverse roles [25]. Previous studies have shown that certain BmSP family members are specifically expressed in the silkworm midgut. For example, BmSP2 plays an important role in defense against *BmNPV* [8], whereas BmSP36 and BmSP141 are primarily involved in food digestion [29,30]. In the humoral immune response of silkworms, BmSPs play crucial roles by participating in the prophenoloxidase (proPO) activation cascade. These proteases convert inactive proPO into active phenoloxidase (PO), which catalyzes the synthesis of quinones and melanin to combat pathogenic microorganisms [31]. BmSPs typically contain a clip domain and possess highly conserved active-site motifs, including TAAHC, DIAL, and GDSGGP [32]. These structural features enable the activation of proPO through cleavage at specific amino acid residues at the carboxyl terminus [33]. Tanaka *et al*. [34] identified multiple BmSPs and BmSPHs involved in silkworm immune responses, some of which are specifically expressed in midgut tissues and primarily function in food digestion under normal conditions. However, upon *BmNPV* infection, the transcriptional levels of these genes are altered. Previous studies have demonstrated that several members of the *BmSP* gene family exhibit induced following expression *BmNPV* infection [35,36]. Given that the midgut serves as the first barrier against *BmNPV* invasion and that certain some midgut-specific genes have established roles in digestion, their precise functions in immune responses remain incompletely understood. Therefore, comprehensive investigation of serine protease genes if of substantial scientific importance.

Following *BmNPV* infection, 18 silkworm serine protease (SP) and serine protease homolog (SPH) genes exhibit varying degrees of upregulation or downregulation. This study focuses on *BmSP25*, a gene that is highly expressed in the midgut and significantly upregulated upon infection [28,37]. First, the differential expression of BmSP25 between the resistant strain SuN and the susceptible strain P50 was analyzed following *BmNPV* infection. Subsequently the effects of BmSP25 overexpression or knockdown on *BmNPV* proliferation were examined. In addition, the expression patterns and subcellular distribution of the BmSP25 protein were analyzed, and potential interacting proteins were screened. Through these approaches, this study systematically elucidates the molecular mechanism by which BmSP25 contributes to resistance against *BmNPV*.

## Materials and methods

### Silkworm rearing and viral particles

The *BmNPV*-susceptible silkworm strain P50 (LC₅₀ = 2.47 × 10⁵ OBs/mL), the *BmNPV*-resistant strain SuN (LC₅₀ = 1.43 × 10⁷ OBs/mL), and the *BmNPV*-Baoshan strain were maintained at the Sericulture and Apiculture Research Institute, Yunnan Academy of Agricultural Science, Honghe, China. Silkworms were reared to the first day of the fifth instar prior to viral infection. Each treatment was conducted in triplicate with 100 larvae per replicate. The experimental group was orally inoculated with 5 μL of *BmNPV* suspension (1.21 × 10⁶ PIB/mL), whereas the control group received an equal volume of ddH₂O. After inoculation, larvae were fed fresh mulberry leaves. Midgut tissues were collected at multiple time points (6 h, 12 h, 24 h, 48 h, 72 h, and 96 h), carefully freed of peritrophic membranes and contents, immediately frozen in liquid nitrogen, and stored at −80℃ until use. A recombinant virus expressing enhanced green fluorescent protein (BV-EGFP) was provided by the State Key Laboratory of Resource Insects, Southwest University, Chongqing, China. The BV-EGFP titer (OBs/mL) was determined using the endpoint dilution method.

### BmN cell culture and transfection

The silkworm ovarian cell line BmN was cultured at 28°C in TC-100 medium supplemented with 10% (v/v) fetal bovine serum and 1% penicillin–streptomycin. Transfections were performed using Cellfectin™ II Reagent (Thermo Fisher Scientific, Waltham, USA) according to the manufacturer’s instructions. Cells were seeded in six-well plates at 60 ～ 80% confluency. Two hours prior to transfection, the culture medium was replaced with serum- and antibiotic-free TC-100 medium following three washes. For preparation of the transfection complexes, 2 μg of plasmid DNA or 20 μM siRNA was mixed with 100 μL of serum-free dilution medium, followed by the addition of 2 μL of transfection reagent and incubation at room temperature for 30 min. The resulting mixture was added to the cell culture wells and gently mixed. Fluorescence images were captured using an Olympus inverted fluorescence microscope (IX73, Olympus, Japan).

### Bioinformatics analysis

The coding sequence of the *BmSP25* gene was obtained from SilkBase (https://silkbase.ab.a.u-tokyo.ac.jp/). The nucleotide and amino acid sequence of BmSP25 were analyzed using DNAMAN 10 (Lynnon Corporation, Quebec, Canada). Conserved motifs were predicted using on the SMART server (http://smart.embl-heidelberg.de/), and signal peptide prediction was performed with SignalP 5.0 (https://services.healthtech.dtu.dk/services/SignalP-5.0/).

### RNA isolation and cDNA synthesis

Total RNA was extracted from silkworm midgut tissues using the Takara MiniBEST Universal RNA Extraction Kit (Takara, Osaka, Japan). RNA concentration and purity (A260/A280 ratio) were determined using a NanoPhotometer N50T (IMPLEN, Munich, Germany), and RNA integrity was assessed by 1% agarose gel electrophoresis. First-strand cDNA was synthesized using the PrimeScript™ RT Reagent Kit with gDNA Eraser (Takara, Osaka, Japan) and stored at −20°C until further analysis.

### Quantitative reverse transcription PCR (RT-qPCR) analysis

RT-qPCR was performed to determine the relative expression levels of target genes using primers listed in Table 1. Reactions were prepared using the SYBR Premix Ex Taq™ II (Tli RNaseH Plus) Kit (Takara, Osaka, Japan) in a 20 μL, containing10 μL SYBR premix, 0.4 μL ROX Reference Dye, 0.4 μL each of forward and reverse primers (10 μmol/L), 1 μL of cDNA template, and 7.8 μL ddH₂O. The thermal cycling conditions were as follows: 95°C for 5 min; followed by 40 cycles of 95°C for 15 s and 60°C for 30 s. Each sample was analyzed in triplicate. Relative gene expression level was calculated using the 2^-ΔΔCt^ method [38], with *BmGAPDH* used as the internal reference gene. Based on the standard curves constructed for the *BmNPV vp39* and *gp41* genes in this study (S1 Fig), absolute quantification was employed to viral copy numbers in different samples.

**Table 1:** Primer used in RT-qPCR.

Primer name	Forward primer (5′-3′)	Reverse primer (5′-3′)
**qBmSP25**	TTCCGTGACACTCTTCAGCG	TGCCGAGGATCCAACAAAGT
**qVP39**	CAACTTTTTGCGAAACGACTT	GGCTACACCTCCACTTGCTT
**qGP41**	CGTAGTAGTAGTAATCGCCGC	AGTCGAGTCGCGTCGCTTT
**qEGFP**	GTCCAGGAGCGCACCATCTT	TTCTGCTTGTCGGCCATGATAT
**qBmGAPDH**	CGATTCAACATTCCAGAGCA	GAACACCATAGCAAGCACGAC

### Prokaryotic expression and antibody preparation

Specific primers for BmSP25 were designed as follow: F: 5′-gatcGGATCCTTCACACTGCCACTGCACGAAAACC-3′ R: 5′-gcAGATCTTTACAGGTGCTGATTGAAGAAGTTC-3′ (underlined sequences indicate *Bam*H I and *Bgl* II restriction sites). The target fragment was amplified using P50 midgut cDNA as the template, cloned into the pMD19-T vector, subsequently subcloned into the pET-30a vector via double digestion. The recombinant plasmid was transformed into *Escherichia. coli* BL21 cells. The recombinant protein was purified using Ni-NTA agarose resin (Qiagen, Hilden, Germany) and used to immunize injected into New Zealand white rabbits for the production of polyclonal antibodies.

### Western blot analysis

Midgut tissues were dissected, and total protein was extracted as previously described [39]. Cells were lysed using lysis buffer (Beyotime, China), washed twice with PBS, and denatured in 5 × SDS-PAGE loading buffer (Beyotime) at 100°C. Protein sample were separated by 12% SDS-PAGE and transferred onto PVDF membranes. Membranes were incubated with primary antibodies (anti-BmSP25, 1:5000; anti-β-actin3, Beyotime, 1:5000) for 1 h at 25°C, followed by incubation with horseradish peroxidase-conjugated secondary antibodies (Beyotime). Protein signals were detected using an ECL Western Blot Detection System (Bio-Rad), and grayscale values were quantified using AlphaEase FC software.

### Effect of *BmSP25* RNAi and overexpression on BV-EGFP proliferation in BmN cells

*BmSP25* targeting siRNAs were synthesized by General Biosystems (Chuzhou, China) and reconstituted to a final concentration of 20 μM using RNase-free H₂O. The experimental groups consisted of BmN cells transfected with *BmSP25* siRNA, whereas the control groups included untransfected cells, cells transfected with negative control siRNA, and cells transfected with DsRed siRNA. After 24 h of incubation with *BmSP25* siRNA, total RNA was extracted and reverse-transcribed into cDNA. RT-PCR was performed to evaluate the RNA interference efficiency of *BmSP25* and to identify the optimal siRNA fragment. Subsequently cells were infected with BV-EGFP and observed at 24, 48, 72, and 96 hour post-infection (hpi). Genomic DNA was extracted, and viral copy numbers were quantified by RT-qPCR targeting the *vp39* gene. For overexpression analysis, the coding sequence of *BmSP25* was amplified using the following primers: F: 5′-gatcGGATCCATGAAGACATTCGTTGCGACC-3′, R:5′-gcGCGGCCGCTTAAAGGTGTTGATTGAAGAAATTC-3′ (underlined sequences indicate *Bam*H I and *Not* I restriction sites). The amplified fragment was first cloned into the pSL1180 intermediate vector and subsequently sub-cloned into the pIZT/V5-His-mCherry vector to generate the recombinant plasmid pIZT/V5-His-mCherry+hr3-*i.e.,1*p-BmSP25. BmN cells were transfected with the constructed plasmid and harvested 48 h later for RNA and DNA extraction. The overexpression efficiency of *BmSP25* was assessed by RT-qPCR, and viral copy numbers were determined as described above.

### Effect of BmSP25 overexpression and knockout on *BmNPV* proliferation in silkworms

The transgenic overexpression vector pBac[*A3*p-EGFP-SV40 + *BmP2*p-BmSP25-SV40] was constructed by inserting the *BmSP25* expression cassette into the pBac[*A3*p-EGFP-SV40] backbone. For gene knockout, sgRNA sequences targeting *BmSP25* were cloned into a U6 promoter-driven vector, and the resulting cassette was inserted into pBac[*A3*p-DsRed-SV40] to generate pBac[*A3*p-DsRed-SV40 + U6-sgRNA-SV40]. The silkworm strain 305 was used for generating transgenic silkworms. Both vectors were co-injected with the helper plasmid pHA3PIG into silkworm eggs (G0). G1 larvae expressing fluorescent markers were selected for subsequent experiment. Total RNA was extracted from the midgut tissues of *BmSP25* overexpressing (*BmSP25*-OE, green fluorescent) and negative control individuals at the fifth instar on day 3, and the transcriptional level of the *BmSP25* gene was analyzed by RT-qPCR. *BmSP25*-OE and wild-type silkworms were orally inoculated at the 5^th^-1 d instar with 5 μL of *BmNPV* (1.21 × 10⁶ PIB/mL) or ddH₂O. Silkworms were maintained on fresh mulberry leaves, and daily mortality was recorded. Midgut tissues were collected at 24, 48, 72, and 96 hpi for DNA extraction and viral DNA quantification by RT-qPCR. For knockout experiments, *BmSP25*-sgRNA transgenic larvae (red fluorescent) were crossed with Cas9-overexpressing larvae (Cas9-OE, green fluorescent). Hybrid offspring positive for both fluorescent markers were selected, and genomic DNA was extracted. PCR amplification and clone sequencing of the target site were performed to evaluate knockout efficiency. The knockout efficiency of *BmSP25* was further assessed by RT-qPCR using midgut tissues from the third day of the fifth instar. Double-fluorescent offspring were selected for viral challenge, and viral replication was evaluated as described above.

### Statistical analysis

Statistical analyses were performed using GraphPad Prism 8. Differences between groups were evaluated using Student’s t-test. A *P* value< 0.05 was considered statistically indicated significant, and a *P* value < 0.01 considered highly significant (*P < 0.05, **P < 0.01, ***P < 0.001). All data were presented as the mean ± SD from at least three independent experiments.

## Results

### Characterization of the BmSP25 sequence

**Fig 1:**
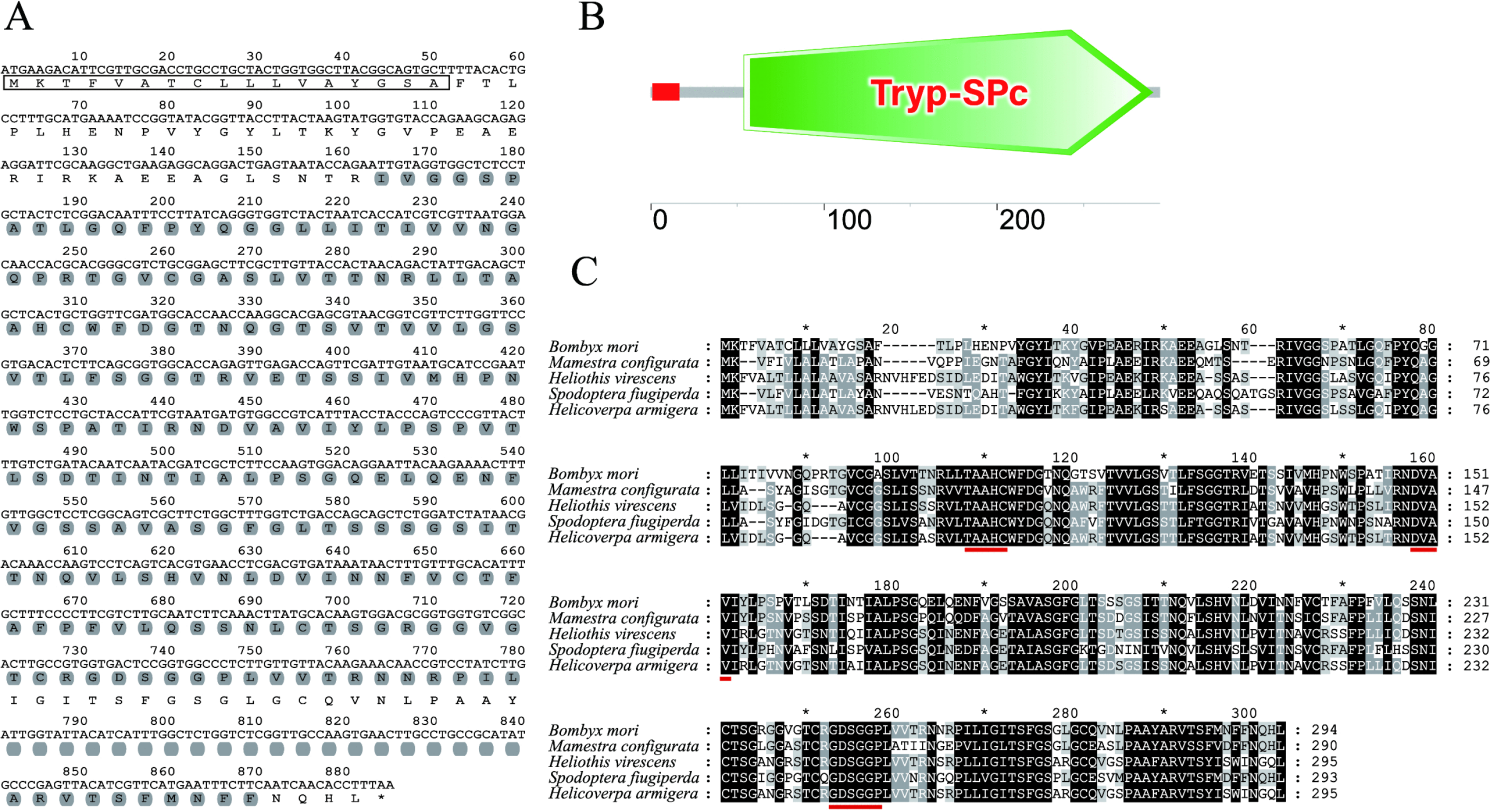
Bioinformatics analysis of BmSP25. (A) The ORF nucleotide sequence of *BmSP25* and its deduced amino acid sequence. BmSP25 protein signal peptide (1–17) was represented by the rectangle. The Tryp_SPc domain is shaded gray. (B) Functional domain prediction of BmSP25 by using SMART online software. (C) Three conserved domains of TAAHC, DVAV and GDSGGPL were labeled the red underline. The GenBank number of each sequence was as follows: *Bombyx mori* (this study), *Mamestra configurata* (ADM35105.1), *Heliothis virescens* (AFM28248.1), *Spodoptera frugiperda* (AIR09775.1), *Helicoverpa armigera* (ADI32882.1).

The cDNA sequence (KWMTBOMO10987) of *BmSP25* contains an open reading frame (ORF) of 885 bp, encoding a protein of 294 amino acids with a predicted molecular weight of 30.86 kDa and an isoelectric point of 8.19. The first 17 amino acid residues at the N-terminus of BmSP25 constitute a signal peptide (positions 1–17), indicating that BmSP25 is a secreted protein (Fig 1A). Conserved domain analysis using SMART software revealed that BmSP25 contains a trysin-like serine protease domain (Tryp_SPc) spanning 54–290 (Fig 1B). Three highly conserved motifs—TAAHC, DVAV, and GDSGGP—were identified in the deduced amino acid (Fig 1C), confirming that BmSP25 belongs to the serine protease family.

### BmSP25 exhibited specific expression patterns in response to *BmNPV* infection

**Fig 2:**
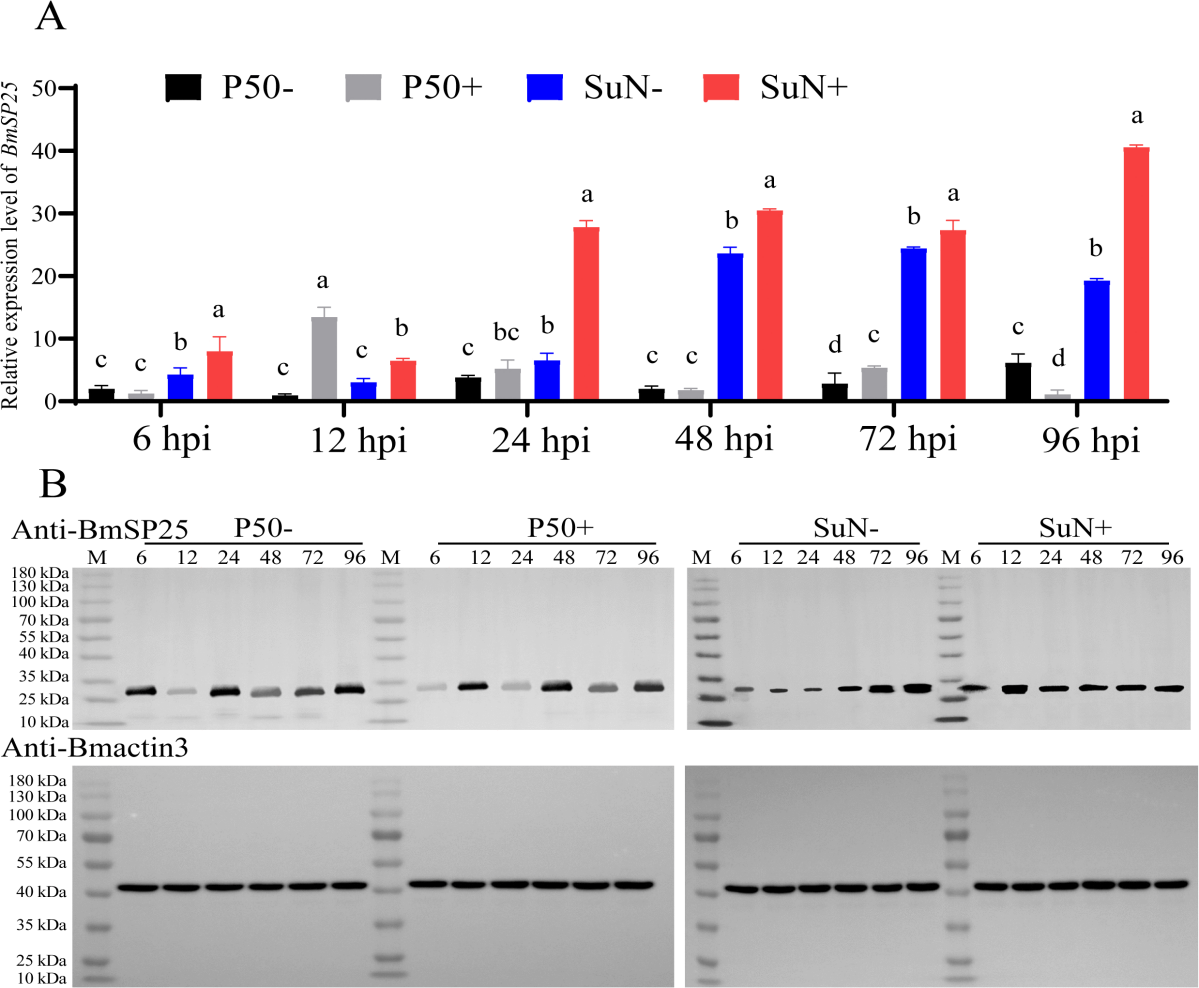
Expression analysis of BmSP25 in silkworm strains SuN and P50 after *BmNPV* infection. (A) RT-qPCR analysis of *BmSP25* mRNA transcriptional levels in SuN and P50 strains; (B) Western blot analysis of BmSP25 protein expression changes in SuN and P50 strains before and after *BmNPV* infection. Significant differences were indicated by different letter (*P* < 0.05). “+” and “-”: *BmNPV*-fed infection group and non-fed control group, respectively.

The dynamic changes in BmSP25 transcription and protein expression in midgut tissues of silkworm strains with different levels of resistance were comparatively analyzed at multiple time points following *BmNPV* infection. In the susceptible strain P50, the transcriptional level of *BmSP25* was upregulated between 6 and 24 hpi. In contrast, in the resistant strain SuN, *BmSP25* expression showed a time-dependent and significant increase throughout the course of infection. Under uninfected conditions, the basal transcriptional level of *BmSP25* in the resistant strain SuN was significantly higher than that in the susceptible strain P50 at all examined time points. Furthermore, the expression difference between the two strains remained significant from 24 to 96 h after infection (Fig 2A). At the protein level, BmSP25 expression in the resistant strain SuN was significantly higher than that in the susceptible strain P50 as early as 24 hpi following *BmNPV* infection, and the overall expression pattern was largely consistent with the transcriptional data (Figs 2B, S2, S1 Table). These findings suggested that BmSP25 had been involved in the immune response of the silkworm midgut to *BmNPV* infection.

### BmSP25 interference affected the susceptibility of BmN cells to *BmNPV*

**Fig 3:**
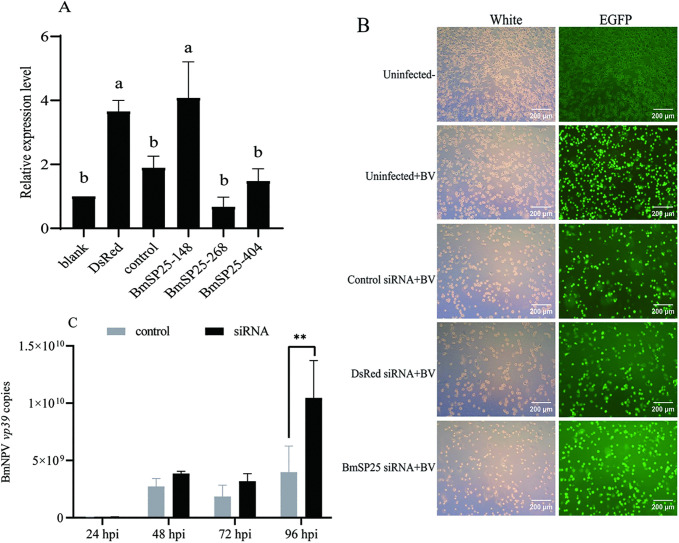
Effect of *BmNPV* transduction in *BmSP25*-RNAi BmN cells. (A) Verification of *BmSP25* gene interference effect; (B) Fluorescence observation of BmN cells infected with BV-EGFP at 72 hpi, scale bar = 200 μm; (C) Analysis of *vp39* expression levels in BmN cells infected with BV-EGFP at different time points, the qPCR employed an absolute quantification method. Significant differences were indicated by different letter (*P* < 0.05). * Indicates significant differences at *P* < 0.05, ** indicates significant differences at *P* < 0.01 with respect to the control.

To investigate the effect of *BmSP25* interference, negative control siRNA and three siRNA fragments targeting specific regions of the *BmSP25* gene were transiently transfected into BmN cells to suppress *BmSP25* expression. Cells were collected 24 hpi, and the expression level of *BmSP25* was analyzed by RT-qPCR. The results showed that the siRNA targeting the *BmSP25*–268 site achieved the most significant interference efficiency, resulting in a markedly lower expression level of the target gene compared with in normal BmN cells (Fig 3A). Subsequently, BmN cells transfected with *BmSP25* siRNA were infected with the recombinant virus BV-EGFP after 24 h. Control groups included cells transfected with DsRed siRNA followed by viral infection, cells infected with BV-EGFP alone (without transfection), and untreated BmN cells (without transfection or viral infection). To assess the impact of *BmSP25* interference on viral infection, viral proliferation was evaluated. As shown in Fig 3B, the number of green fluorescence-positive cells in the *BmSP25* interference group was significantly higher than that in the control groups. Consistently, the expression level of the viral gene *vp39* in the *BmSP25* interference group was significantly upregulated compared with that in normal BmN cells (Fig 3C). These results indicate that suppression of *BmSP25* expression in BmN cells facilitates *BmNPV* infection.

### *BmSP25* overexpression enhanced the susceptibility of BmN cells to *BmNPV* invasion

**Fig 4:**
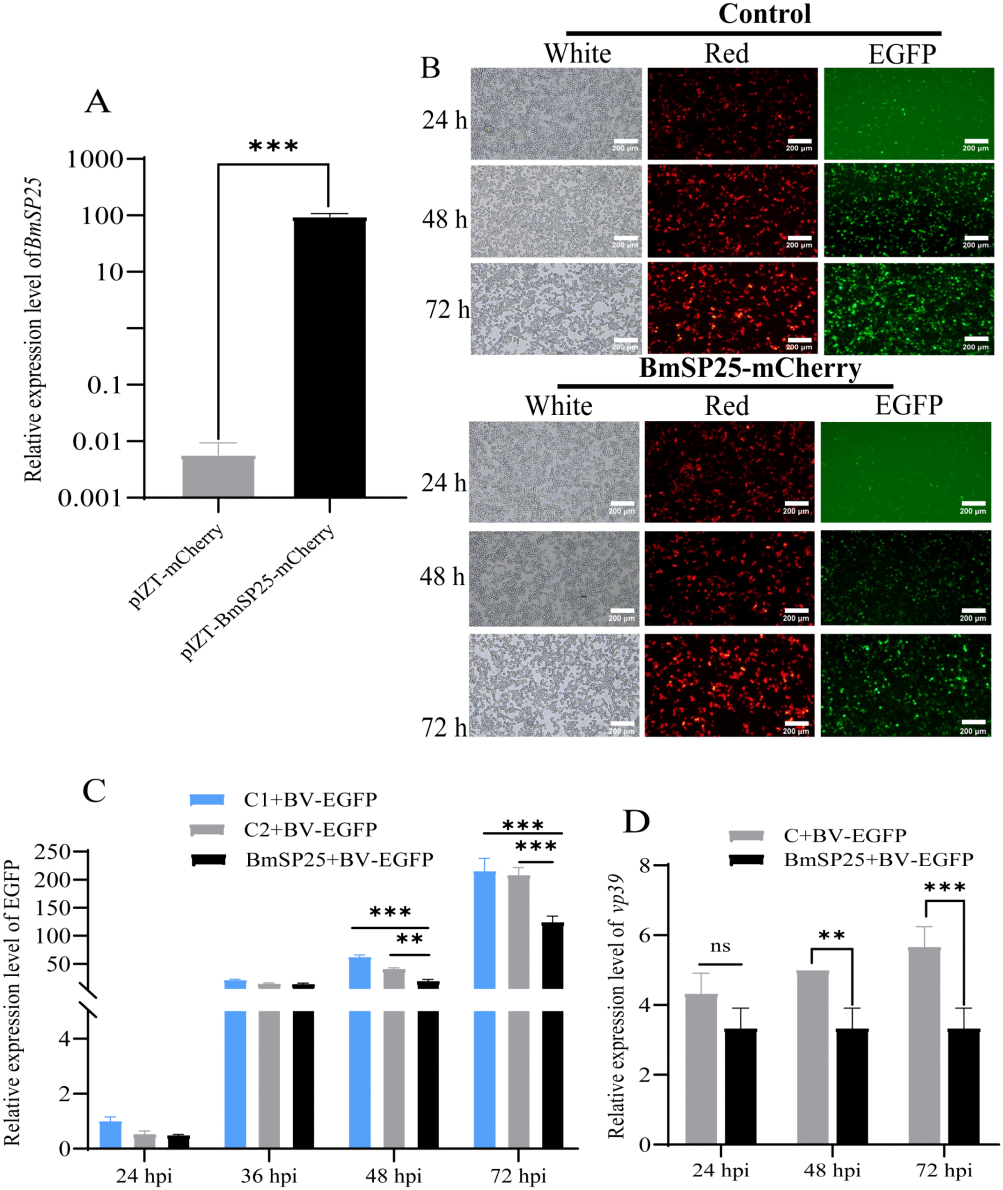
Effect of *BmNPV* transduction in BmSP25 overexpression BmN cells. (A) The transcriptional level of BmSP25 in BmN cells transfected with pIZT-hr3-*i.e.,1*p-BmSP25/V5-His-mCherry at 48 h after transfection; (B) Green fluorescence in cells at 48 hpi with *BmNPV*, Scale bar = 200 μm; (C) Analysis of EGFP expression in BmN cells 24, 36, 48 and 72 hpi with *BmNPV*, C1 represented normal BmN cells infected with BV-EGFP, while C2 represented BmN cells transfected with the pIZT/V5-His-mCherry vector that did not contain the *BmSP25* gene; (D) Analysis of *vp39* expression in BmN cells 24, 48 and 72 hpi after infection with *BmNPV*, the qPCR employed a relative quantification method; C: BmN + pIZT/V5-His-mCherry vector. Significant differences are indicated by asterisks (*P < 0.05; **P < 0.01; ***P < 0.001), ns: no significant.

Successful overexpression of *BmSP25* in BmN cells was confirmed by RT-qPCR analysis (Fig 4A), BmN cells overexpressing *BmSP25* were subsequently infected with the recombinant virus BV-EGFP at 48 hpi. Normal BmN cells served as the uninfected control. Two experimental groups were established: BmN cells infected with BV-EGFP alone and BmN cells transfected with either pIZT-hr3-*i.e.,1*p-*BmSP25*/V5-His-mCherry or pIZT/V5-His-mCherry vector followed by BV-EGFP infection. At 48 hpi, fluorescence microscopy and RT-qPCR were performed to evaluate the expression levels of EGFP and *vp39*. Red fluorescence was observed in both *BmSP25*-overexpressing cells (BmN^pIZT-hr3-*i.e.,1*p-*BmSP25*/V5-His-mCherry^) and vector control cells (BmN^pIZT/V5-His-mCherry^) (Fig 4B). Green fluorescence signals were detected at multiple time points following viral infection. At 48 and 72 hpi, the green fluorescence intensity in *BmSP25*-overexpressing cells was notably weaker than that in control cells. RT-qPCR analysis demonstrated that EGFP expression at 48 hpi was significantly lower in *BmSP25*-overexpressing cells than in control cells (Fig 4C). Similarly, *vp39* expression was significantly reduced in *BmSP25*-overexpressing BmN cells compared to controls (Fig 4D). In addition, the number of viral particles in the culture supernatant of *BmSP25*-overexpressing BmN cells was significantly lower than that in the control group. Collectively, these results demonstrate that *BmSP25* overexpression suppresses *BmNPV* proliferation in BmN cells.

### BmSP25 overexpression inhibited the invasion of *BmNPV* into silkworm individuals

**Fig 5:**
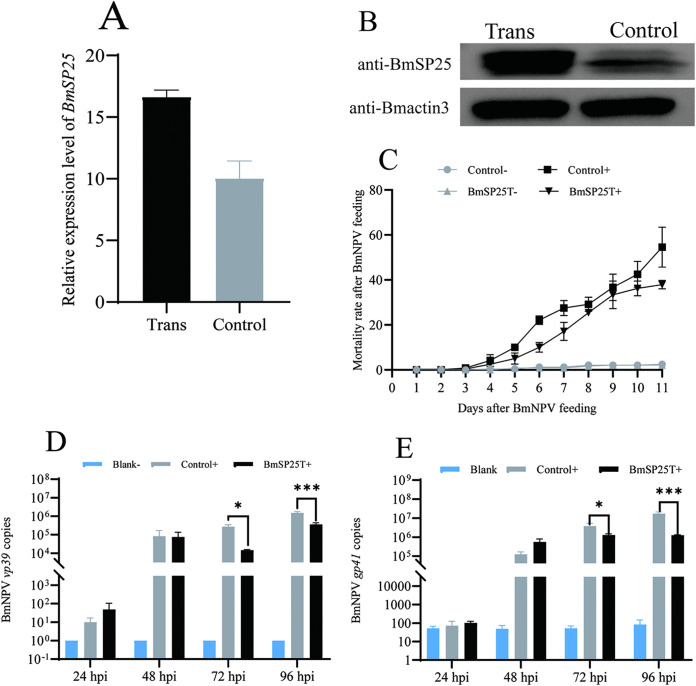
The effect of transgenic silkworms overexpressing BmSP25 on the proliferation of *BmNPV.* (A) Analysis of transcription levels of *BmSP25* between BmSP25-OE strain and negative individuals; (B) Western Blot Analysis of BmSP25 protein expression between transgenic silkworm and negative individuals; (C) Statistical mortality rate of silkworm larvae after oral administration of *BmNPV*; (D) The copies of *BmNPV vp39* gene; (E) The copies of *BmNPV gp41* gene; “+” and “-”: *BmNPV*-fed infection group and non-fed control group, respectively. Significant differences are indicated by asterisks (*P < 0.05; ***P < 0.001).

A BmSP25-OE transgenic silkworm strain was generated using a piggyBac transposon vector. Under fluorescence microscopy, ubiquitous green fluorescence was observed in positive transgenic individuals (S3 Fig). RT-qPCR analysis revealed that the expression level of *BmSP25* in the midgut tissues of positive transgenic individuals was significantly higher than that in negative individuals (Fig 5A). To further evaluate the effect of *BmSP25* overexpression on *BmNPV* infection, the first day of the fifth-instar larvae were starved for 24 h, after which positive transgenic positive individuals and negative controls were orally inoculated with occlusion-derived virus (ODV). Mortality was continuously monitored and recorded. The cumulative mortality rates in the non-inoculated negative and positive control groups were 2.5% and 2.1%, respectively, with no significant difference observed. Following after viral inoculation, however, the cumulative mortality rate reached 54.58% in the negative experimental group and 37.92% in the positive experimental group, indicating a significantly enhanced resistance to *BmNPV* infection in *BmSP25*-overexpressing silkworms (Fig 5B). The transcriptional levels of the *BmNPV* genes *vp39* and *gp41* in midgut tissues were measured at 24, 48, 72, and 96 hpi. Viral copy numbers were calculated based on the expression levels of *vp39* and *gp41*. RT-qPCR analysis showed that at 72 and 96 hpi, the transcriptional levels of both *vp39* and *gp41* were significantly lower in the BmSP25 transgenic strain were than in negative controls (Fig 5C, 5D). These results indicated that *BmSP25* overexpression in vivo effectively inhibited *BmNPV* proliferation and replication.

### BmSP25 knockdown enhanced the invasion of *BmNPV* into silkworm individuals

**Fig 6:**
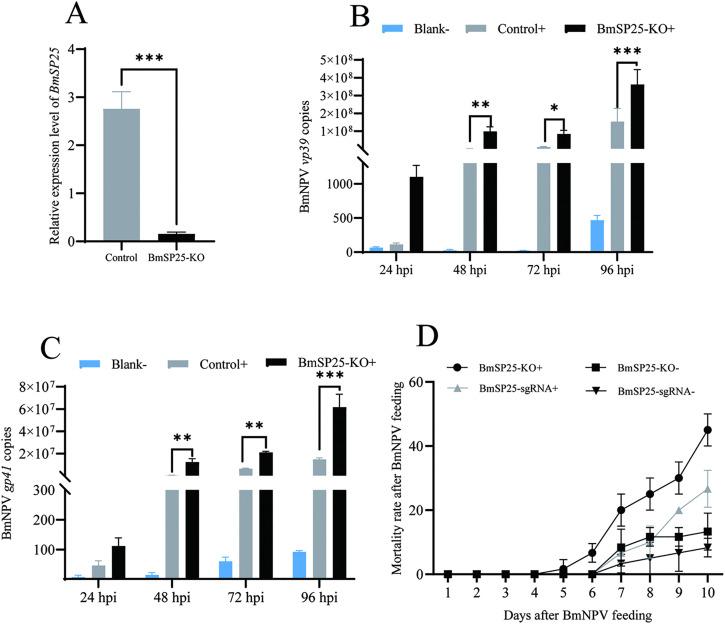
The impact of the *BmSP25*-KO knockout strain of silkworm on the proliferation of *BmNPV.* (A) Analysis of transcription levels of BmSP25 in *BmSP25*-KO strain and negative individuals; (B) The copies of *BmNPV vp39* gene; (C) The copies of *BmNPV gp41* gene; (D) Statistical mortality rate of silkworm larvae after oral administration of *BmNPV*; “+” and “-”: *BmNPV*-fed infection group and non-fed control group, respectively. Significant differences are indicated by asterisks (*P < 0.05; **P < 0.01; ***P < 0.001).

The *BmSP25* knockout hybrid (BmSP25-sgRNA × Cas9) exhibited both red and green fluorescence (S4 Fig). Sequencing analysis confirmed that the two target sites within the *BmSP25* gene were edited by Cas9, resulting in various base deletions and mutations (S5 Fig), thereby verifying the successful generation of targeted knockout line. In *BmSP25* knockout silkworms (*BmSP25*-KO), the expression level of the target gene in midgut tissues of positive individuals was significantly lower than that in negative individuals (Fig 6A). To further assess the effect of *BmSP25* knockout on silkworm resistance to *BmNPV*, negative control individuals and *BmSP25*-KO silkworms were reared under normal conditions until the onset of the fifth instar and then orally inoculated with *BmNPV*. Using standard curves established for the *BmNPV vp39* and *gp41* genes, the transcriptional levels of these viral genes in midgut tissues were measured at 24, 48, 72, and 96 hpi, and viral copy numbers were calculated accordingly. RT-qPCR results showed that the transcriptional levels of both *vp39* and *gp41* in the *BmSP25*-KO strain were significantly higher than those in negative controls (Fig 6B, 6C). In addition, the cumulative mortality rate of the *BmSP25*-KO strain was higher than that of the *BmSP25*-sgRNA strain (Fig 6D). These findings demonstrate that knockdown of BmSP25 in vivo enhances *BmNPV* proliferation and replication, further confirming the critical role of BmSP25 in the host response to *BmNPV* infection.

## Discussion

Serine proteases are a class of endopeptidases characterized by the presence of a serine residue at their active site and perform diverse functions primarily related to development, digestion, and immunity [28,33,40]. In insects, serine proteases and their homologs (SPs/SPHs) are mainly involved in developmental processes and immune responses, playing central roles in immune defense networks—including the melanization cascade, prophenoloxidase activation, and the production of cytokine-like signaling molecules. BmSP25 contains a Tryp_SPc domain, suggesting its potential involvement in food digestion in silkworms [37]. In the present study, we first observed that *BmNPV* infection induced the upregulation of BmSP25, with both basal and induced expression levels being higher in the resistant SuN strain than in the susceptible P50 strain. This expression pattern strongly suggests that BmSP25 may act as an important response factor in silkworm defense against *BmNPV*. To verify its function, gain- and loss-of-function experiments were conducted in BmN cells. The results demonstrated that knockdown of BmSP25 promoted viral proliferation, whereas its overexpression significantly suppressed viral replication. This bidirectional evidence clearly establishes the antiviral function of BmSP25 at the cellular level.

Previous studies have shown that certain silkworm digestive enzymes participate in defense responses against *BmNPV* and that overexpression of endogenous antiviral genes can enhance antiviral capacity. In this study, *BmSP25* was specifically overexpressed in the silkworm midgut using a piggyBac transgenic vector driven by the BmP2 promoter. Overexpression of *BmSP25* resulted in a reduction in viral DNA copies in BmN cells, indicating that, similar to other antiviral proteins, BmSP25 can inhibit *BmNPV* replication. The outer structure of *BmNPV* mainly consists of the capsid and envelope proteins [41]. After incubation with recombinant BmTA and BmSP142 proteins [19,42], viral DNA copy numbers in silkworm larvae and BmN cells were significantly reduced. It has been hypothesized that these proteins may induce proteolytic cleavage of viral structural proteins, thereby disrupt viral integrity and reducing infectivity. Although the present study did not examine the effects of co-incubating recombinant BmSP25 with *BmNPV* virions either *in vivo* or *in vitro*, the high similarity in functional domains and active sites among BmSP25, BmSP142, and BmTA suggests that BmSP25 may mediate antiviral effects through a comparable proteolytic mechanism.

This study revealed an immune-related phenotype of the *BmSP25* gene in defense against *BmNPV* infection in silkworms. Although *BmSP25* has primarily been annotated as a digestion-related gene, its altered expression following viral infection and its regulatory effects on viral proliferation suggest that *BmSP25* may belong to a class of “dual-function” genes in insects that are involved in both metabolism and immune regulation. Similar phenomena have been reported in *Drosophila*, in which certain digestive enzyme genes have evolved to acquire pathogen-recognition or immunomodulatory functions through structural adaptations [43]. In *Drosophila*, clip-domain serine proteases, such as Persephone, Grass, Spirit, and SPE (Spätzle-processing enzyme), participate in activation of the Toll immune signaling pathway, ultimately inducing the expression of antimicrobial peptides. These proteases contain trypsin-like serine protease domains [44], which not only degrade ingested proteins but also play key roles in immune signal transduction. The precursor Spätzle protein is cleaved by serine proteases in the hemolymph to generate active Spätzle, which subsequently binds to the membrane receptor Toll, thereby activating the Toll immune signaling pathway [45–47]. Therefore, BmSP25 may represent an evolutionary adaptation in insects, reflecting a “digestion–immunity coupling” defense strategy that is particularly suited for protection against orally acquired pathogens.

The *BmSP25* gene was knocked out in silkworms using transgenic technology. Guided by *BmSP25*-sgRNA, the Cas9 protein successfully cleaved the target site of the *BmSP25* gene. Compared with normal silkworms, the *BmSP25*-KO strain exhibited significantly higher expression of the viral proliferation-related gene *vp39* at 48 h after oral inoculation with *BmNPV*, indicating that disruption of BmSP25 promoted viral replication in the host. Furthermore, analysis of cumulative mortality throughout the entire fifth instar stage showed that the mortality rate of the *BmSP25*-KO strain was higher than that of normal silkworms, suggesting that loss of BmSP25 reduced the host defense capacity against *BmNPV*. However, the difference in mortality between the experimental and control groups did not reach statistical significance. Possible explanations include: (I) the editing efficiency or mutation type at the selected knockout target site may not have completely disrupted gene function; (II) some mutations resulted in frameshifts that were multiples of three, potentially allowing residual or truncated protein function; and (III) functional compensation by other genes following loss of BmSP25 may have masked significant changes in resistance in conventional mortality assays. Through both cellular- and individual-level overexpression and knockdown experiments, we found that overexpression of *BmSP25* significantly reduced *BmNPV* replication efficiency, whereas interference with or knockout of the gene increased viral susceptibility. These results indicate that *BmSP25* plays a critical role in antiviral immunity. This study not only identifies *BmSP25* as a novel key gene involved in silkworm resistance to *BmNPV* but also confirms its important function through multi-level experimental evidence. Collectively, these findings provide new insights into the immune defense mechanisms of silkworms against DNA viruses and establish a theoretical basis, as well as a valuable candidate target, for breeding highly disease-resistant silkworm varieties through gene editing or molecular marker-assisted selection.

## Supporting information

S1 Fig The standard curve of *vp39* and *gp41* gene. (TIF)

S2 Fig The densitometric intensity of the western blot was analyzed by using AlphaEase FC software. Significant differences were indicated by different letter (*P* < 0.05).
(TIF)

S3 Fig Images of positive transgenic silkworm of adults between bright and green lights. Positive individuals show green fluorescence in the silkworm body, scale bar = 2 mm.
(TIF)

S4 Fig Observation of different fluorescence in larvae of the silkworm BmSP25-KO knockout strain. The BmSP25-KO strain exhibited both red and green fluorescence, the BmSP25-sgRNA strain showed red fluorescence, and the Cas9 strain displayed green fluorescence, the negative individual silkworms didn’t carry these two fluorescent markers, scale bar = 2 mm.
(TIF)

S5 Fig DNA sequencing analysis of the CRISPR/Cas9 editing target genes. 305C/WT: Negative individual; sgRNA: Individual containing BmSP25 sgRNA, site1: Knockout site 1 of *BmSP25* gene exon1, site2: Knockout site 1 of *BmSP25* gene exon4; Cas9: Individual expressing only Cas9 protein; KO 1# ～ 10#: Double-fluorescent individual.
(TIF)

S1 Table Gray value of the specimen. (DOCX)

S1 Data Original data. (ZIP)
