# Supplementary figures and images for: Identification of BmSP25 gene in Bombyx mori with antiviral function against BmNPV
Source: PLoS One. 2026 Mar 27;21(3):e0345502. doi: 10.1371/journal.pone.0345502 (PMC13028367; doi:10.1371/journal.pone.0345502)

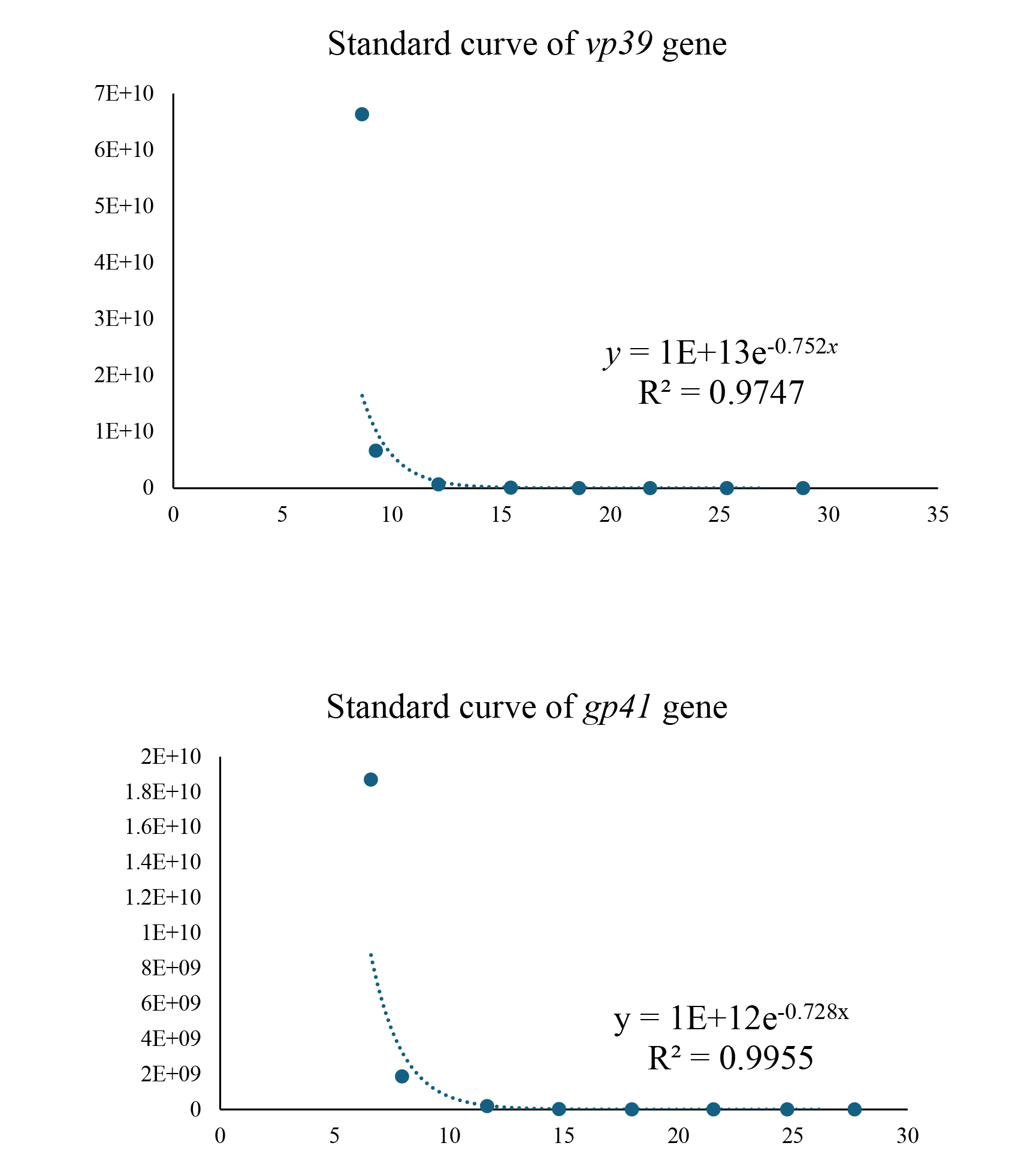

Supplement: S1 Fig — (TIF) [file pone.0345502.s001.tif]

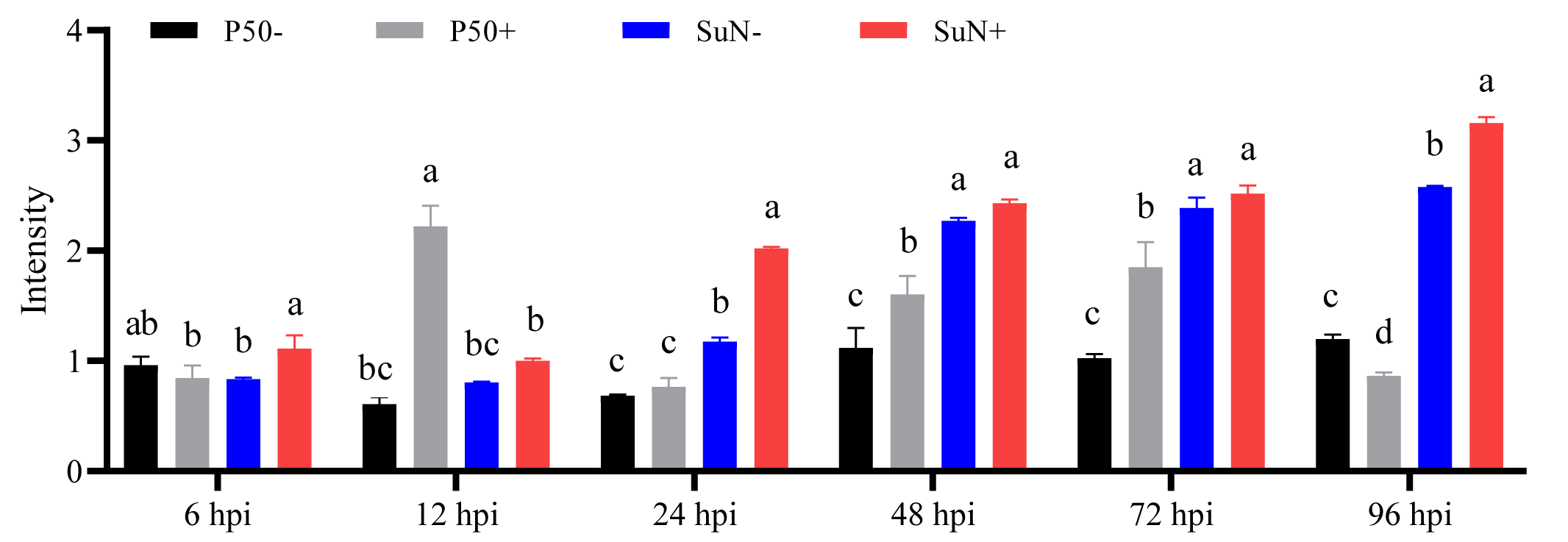

Supplement: S2 Fig — Significant differences were indicated by different letter (P < 0.05). (TIF) [file pone.0345502.s002.tif]

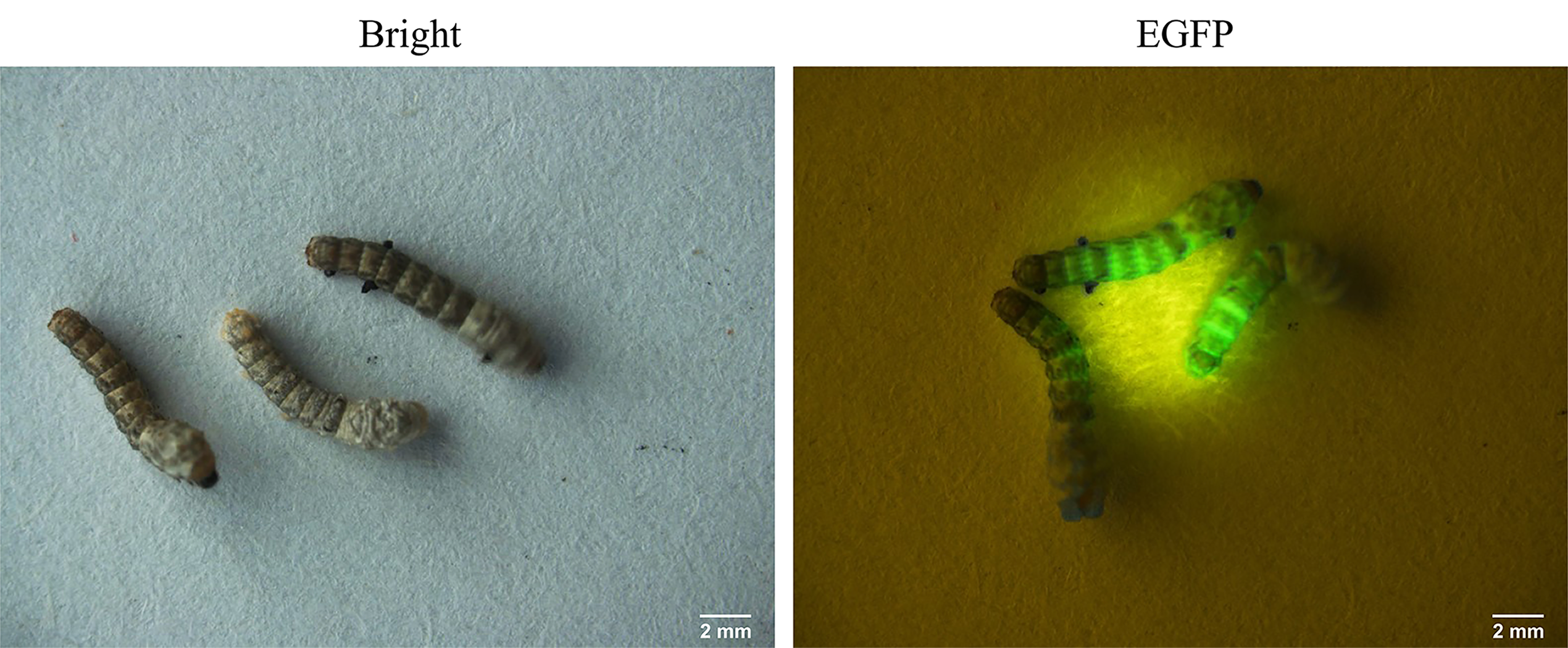

Supplement: S3 Fig — Positive individuals show green fluorescence in the silkworm body, scale bar = 2 mm. (TIF) [file pone.0345502.s003.tif]

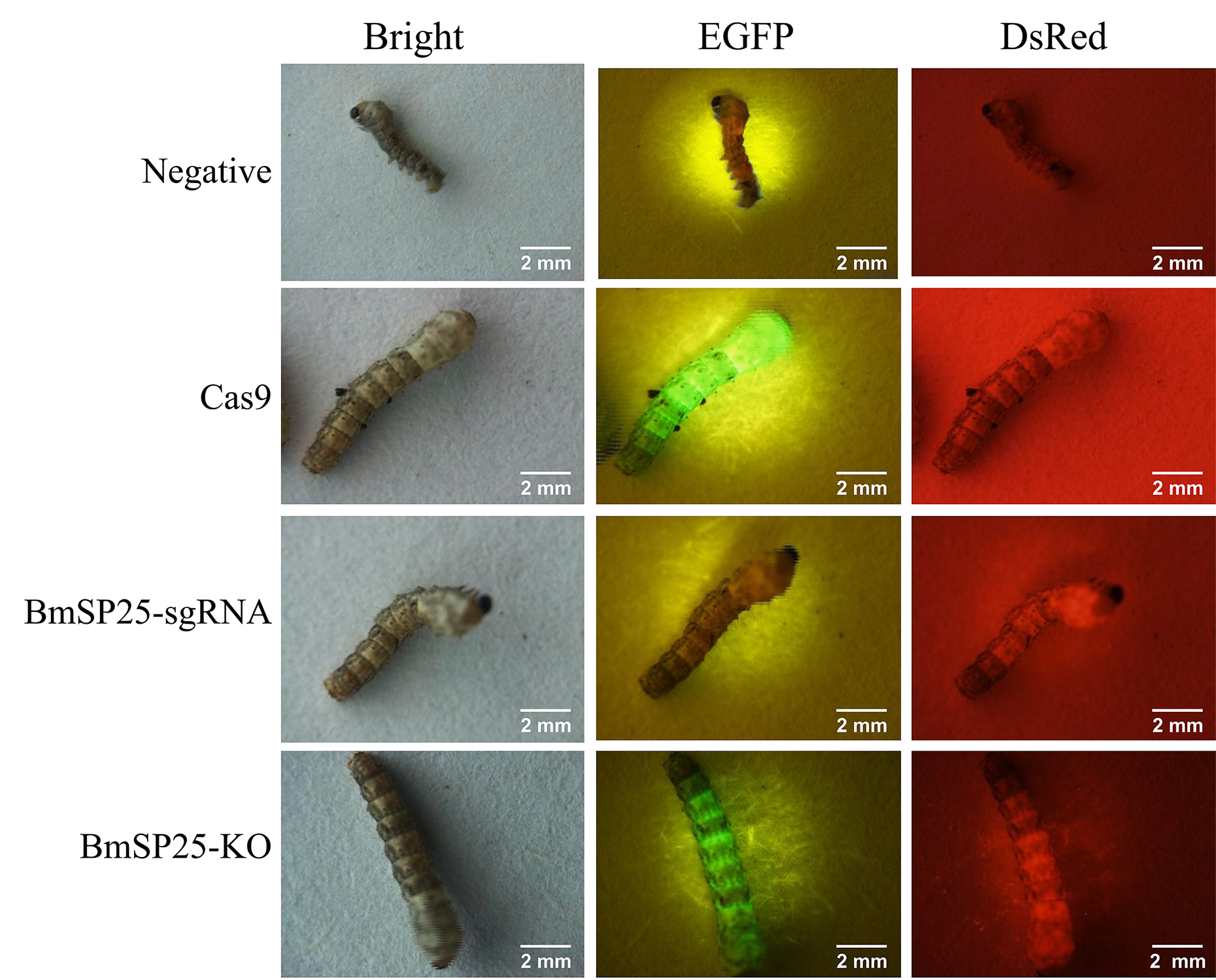

Supplement: S4 Fig — The BmSP25-KO strain exhibited both red and green fluorescence, the BmSP25-sgRNA strain showed red fluorescence, and the Cas9 strain displayed green fluorescence, the negative individual silkworms didn’t carry these two fluorescent markers, scale bar = 2 mm. (TIF) [file pone.0345502.s004.tif]

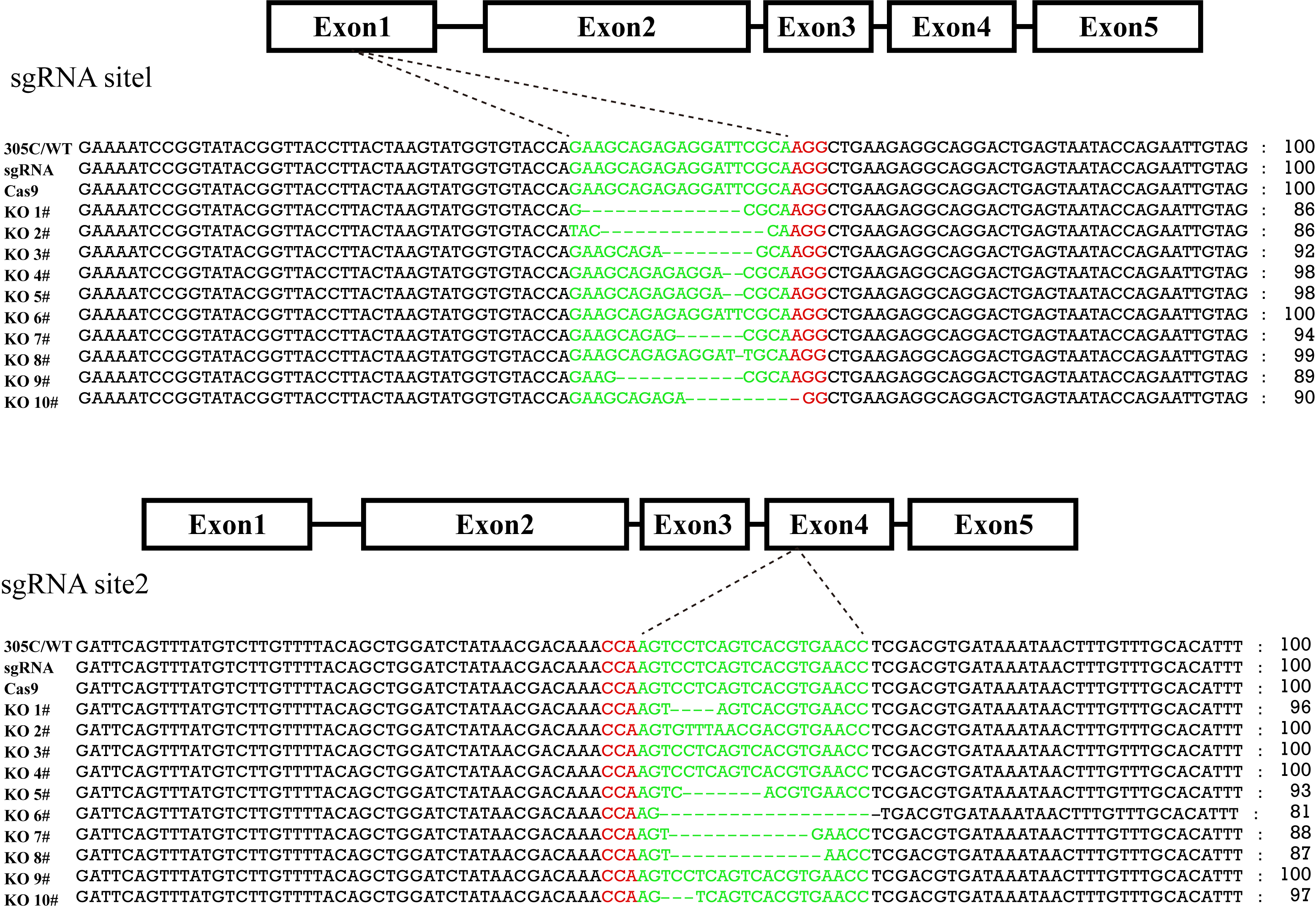

Supplement: S5 Fig — 305C/WT: Negative individual; sgRNA: Individual containing BmSP25 sgRNA, site1: Knockout site 1 of BmSP25 gene exon1, site2: Knockout site 1 of BmSP25 gene exon4; Cas9: Individual expressing only Cas9 protein; KO 1# ～ 10#: Double-fluorescent individual. (TIF) [file pone.0345502.s005.tif]

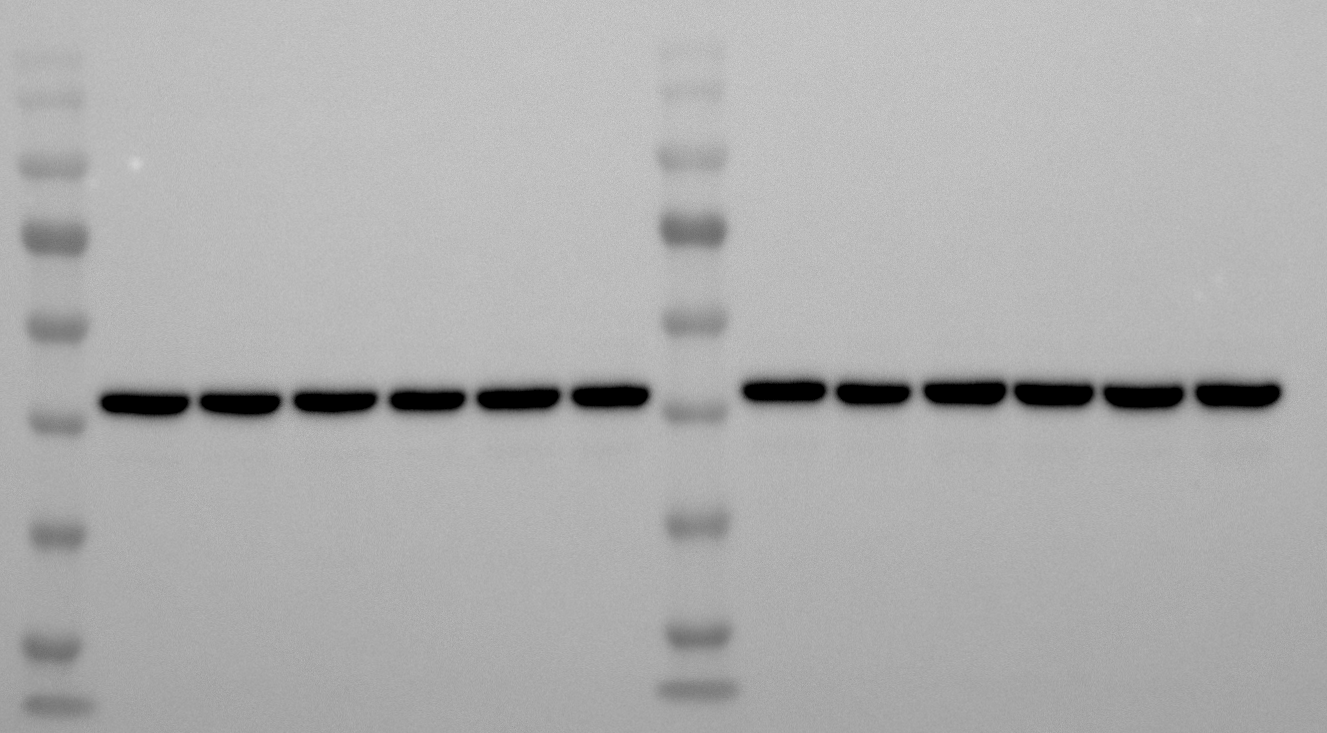

Supplement: S1 Data — (ZIP) [file pone.0345502.s007.zip › original data/WB-P50 anti-Bmactin3 (Fig 2B).tif]

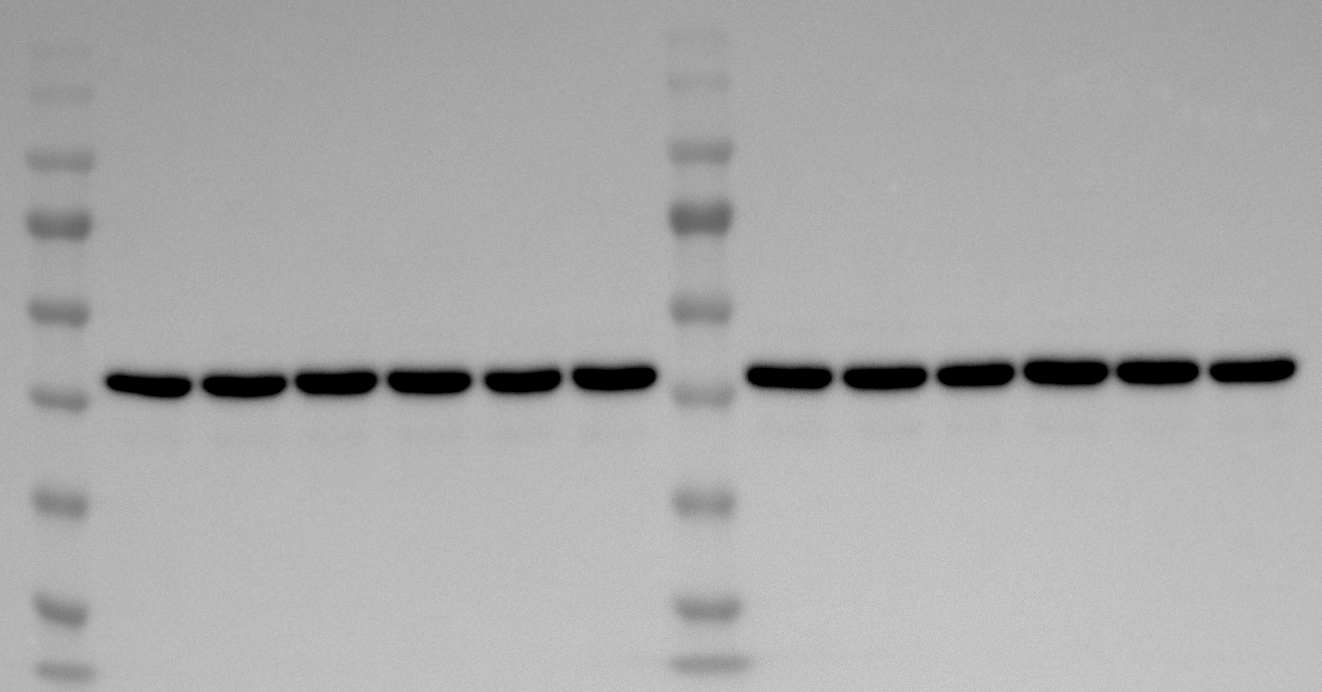

Supplement: S1 Data — (ZIP) [file pone.0345502.s007.zip › original data/WB-SuN anti-Bmactin3 (Fig 2B).tif]

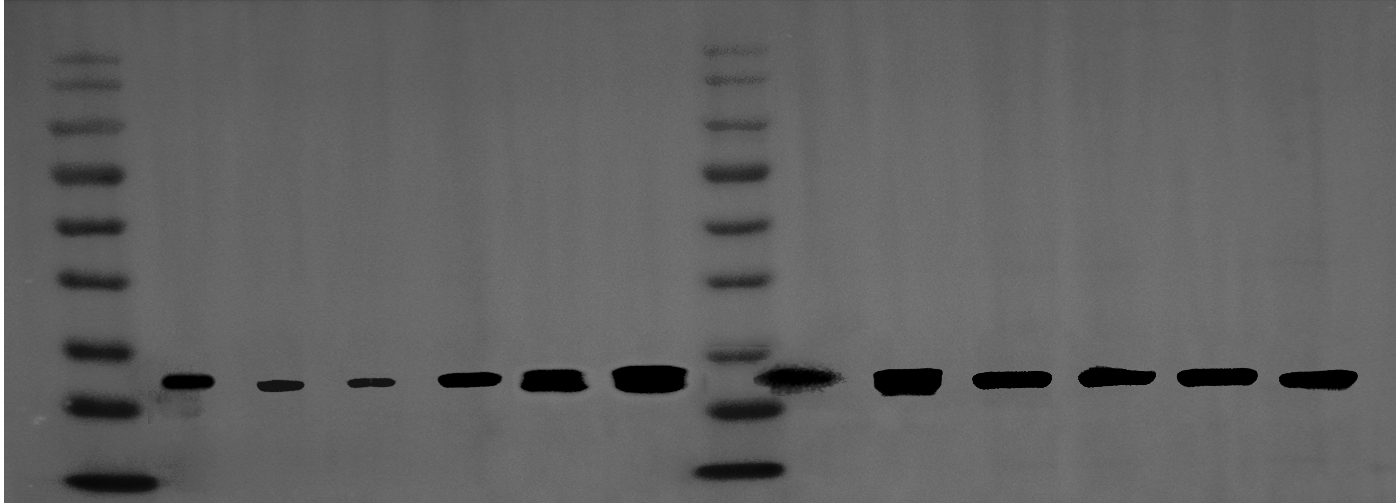

Supplement: S1 Data — (ZIP) [file pone.0345502.s007.zip › original data/WB-SuN anti-BmSP25 (Fig 2B).tif]

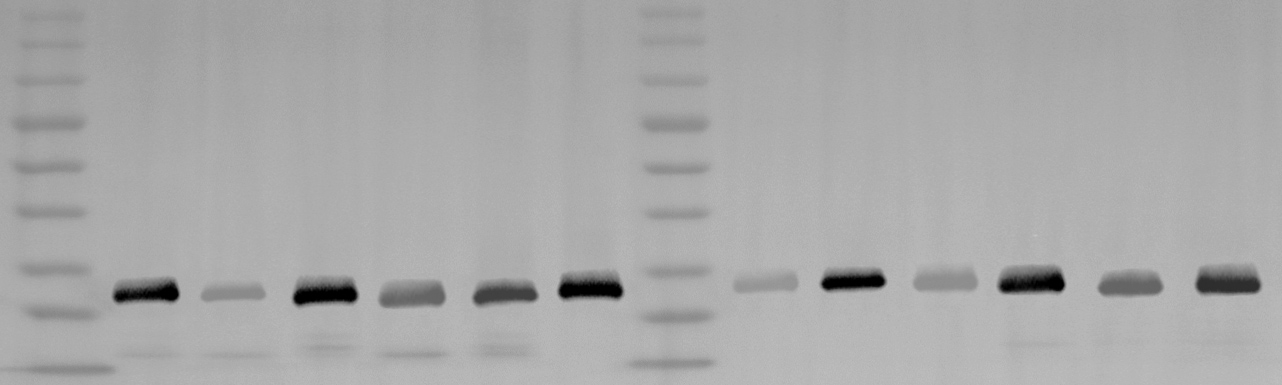

Supplement: S1 Data — (ZIP) [file pone.0345502.s007.zip › original data/WB-P50 anti-BmSP25 (Fig 2B).tif]

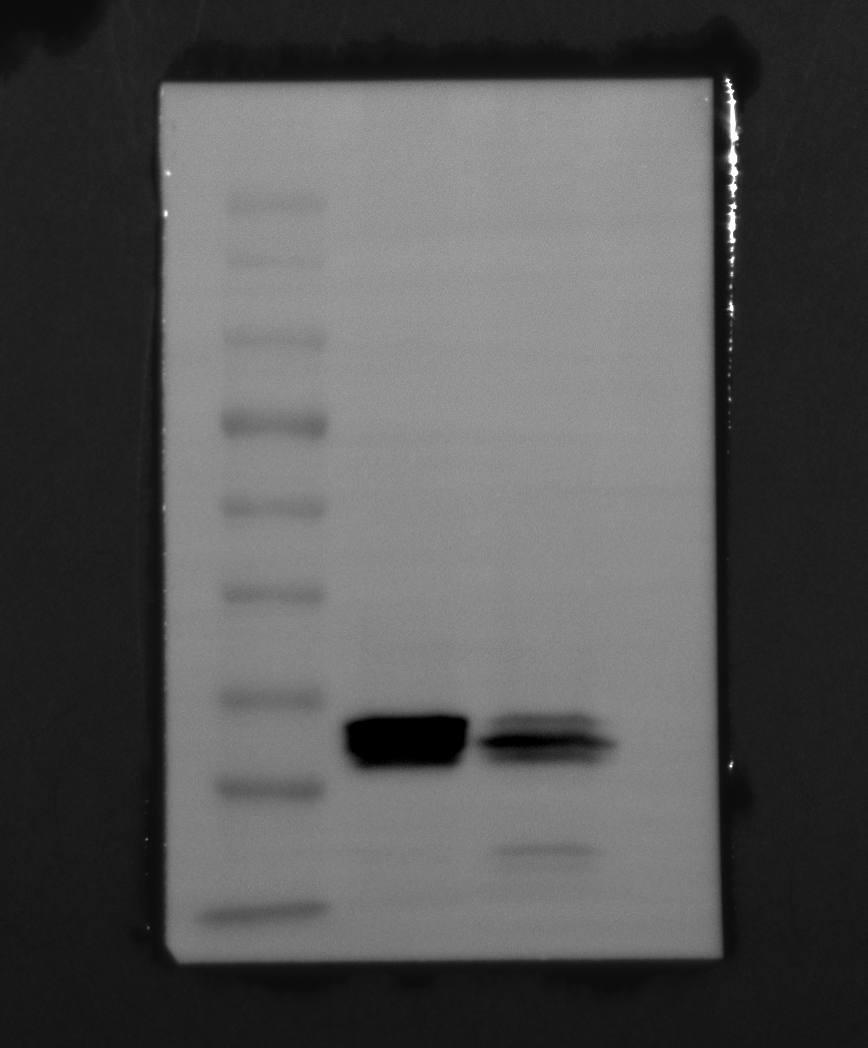

Supplement: S1 Data — (ZIP) [file pone.0345502.s007.zip › original data/BmSP25T WB-anti-BmSP25.tif]

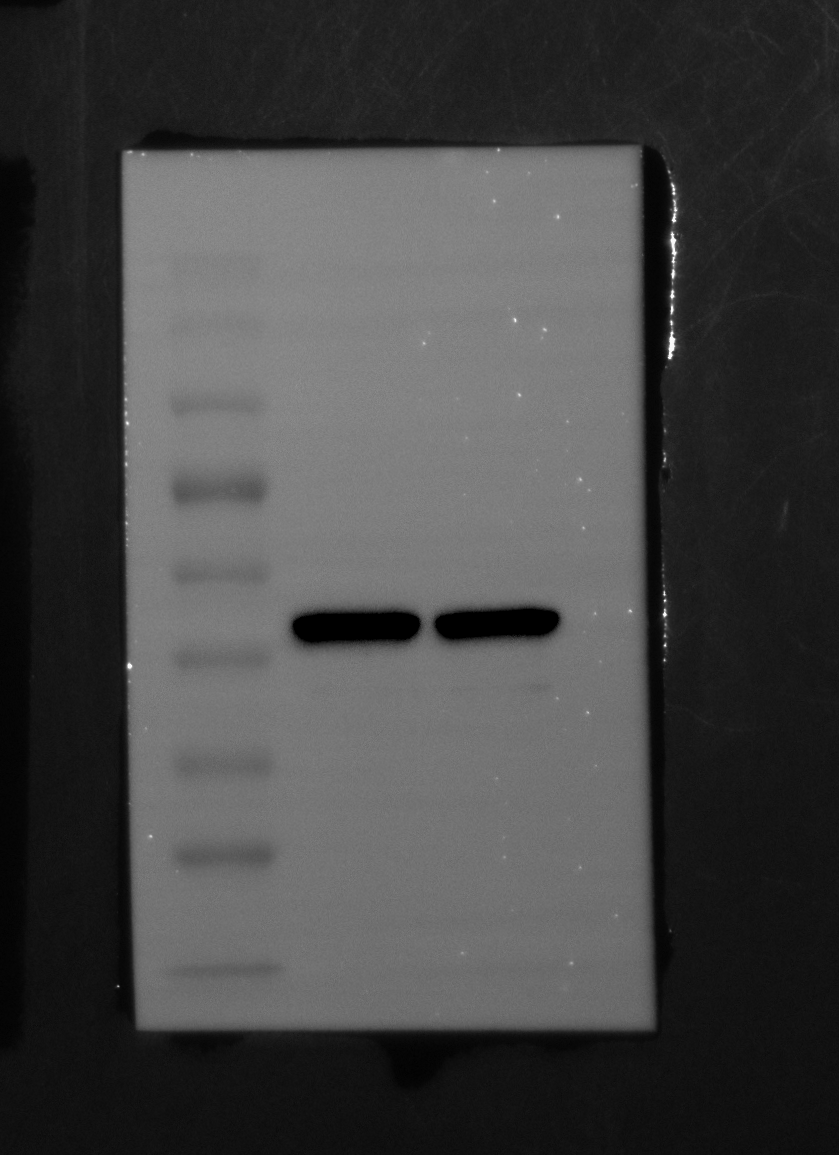

Supplement: S1 Data — (ZIP) [file pone.0345502.s007.zip › original data/BmSP25T WB-anti-Bmactin-3.tif]
