# Supplementary material for: Identification of BmSP25 gene in Bombyx mori with antiviral function against BmNPV
Source: PLoS One. 2026 Mar 27;21(3):e0345502. doi: 10.1371/journal.pone.0345502 (PMC13028367; doi:10.1371/journal.pone.0345502)
Supplement: S1 Table — (DOCX) [file pone.0345502.s006.docx]

**Table S1. Gray value of the specimen**

| Gray value of the specimen (measured using AlphaEase FC software) | | | | | | | | | | | | | |
| --- | --- | --- | --- | --- | --- | --- | --- | --- | --- | --- | --- | --- | --- |
| The value of band gray | | | | | | | | |  | Ratio of the target band gray value to the internal reference band gray value | | | |
| Sample (**P50**) | Bmactin3 | | | Mean value | BmSP25 | | | Mean value |  |  | ΔBmSP25 | | |
| P50/C M-6h | 261123 | 261088 | 261114 | 261108.3333 | 272532 | 233411 | 246751 | 250898 |  | P50/C M-6h | 1.043692053 | 0.893993596 | 0.944993375 |
| P50/C M-12h | 285612 | 289600 | 274532 | 283248 | 185423 | 155628 | 176001 | 172350.6667 |  | P50/C M-12h | 0.649212918 | 0.537389503 | 0.641094663 |
| P50/C M-24h | 314512 | 310852 | 311314 | 312226 | 219711 | 210101 | 210644 | 213485.3333 |  | P50/C M-24h | 0.698577479 | 0.675887561 | 0.676628741 |
| P50/C M-48h | 297903 | 296904 | 295964 | 296923.6667 | 274211 | 376112 | 345744 | 332022.3333 |  | P50/C M-48h | 0.920470757 | 1.266779835 | 1.168196132 |
| P50/C M-72h | 298324 | 298611 | 298485 | 298473.3333 | 316120 | 295963 | 307201 | 306428 |  | P50/C M-72h | 1.059653263 | 0.991132276 | 1.029200797 |
| P50/C M-6h | 297626 | 297780 | 297126 | 297510.6667 | 343624 | 360623 | 365602 | 356616.3333 |  | P50/C M-96h | 1.15454967 | 1.21103835 | 1.230461151 |
| P50/NPV M-6h | 291632 | 294613 | 295711 | 293985.3333 | 211741 | 251653 | 281852 | 248415.3333 |  | P50/NPV M-6h | 0.72605544 | 0.854181587 | 0.953133296 |
| P50/NPV M-12h | 324076 | 312348 | 313111 | 316511.6667 | 786901 | 677004 | 648131 | 704012 |  | P50/NPV M-12h | 2.428137227 | 2.167467056 | 2.069971991 |
| P50/NPV M-24h | 305641 | 301256 | 307452 | 304783 | 257410 | 232614 | 210641 | 233555 |  | P50/NPV M-24h | 0.842197218 | 0.772147277 | 0.685118327 |
| P50/NPV M-48h | 114540 | 112789 | 120056 | 115795 | 161912 | 192845 | 203104 | 185953.6667 |  | P50/NPV M-48h | 1.413584774 | 1.709785529 | 1.691743853 |
| P50/NPV M-72h | 324568 | 337841 | 332357 | 331588.6667 | 658160 | 537894 | 641540 | 612531.3333 |  | P50/NPV M-72h | 2.027803111 | 1.592151337 | 1.930273772 |
| P50/NPV M-96h | 328417 | 328621 | 328457 | 328498.3333 | 286213 | 274425 | 293432 | 284690 |  | P50/NPV M-96h | 0.871492645 | 0.835080534 | 0.893365037 |
| Sample (**SuN**) |  |  |  |  |  |  |  |  |  |  |  |  |  |
| SuN/C M-6h | 345816 | 345124 | 345451 | 345463.6667 | 291632 | 282231 | 290012 | 287958.3333 |  | SuN/C M-6h | 0.84331552 | 0.817766948 | 0.839517037 |
| SuN/C M-12h | 338120 | 338160 | 338170 | 338150 | 269268 | 274123 | 272356 | 271915.6667 |  | SuN/C M-12h | 0.796368153 | 0.810631062 | 0.805381909 |
| SuN/C M-24h | 307260 | 317412 | 305627 | 310099.6667 | 366528 | 359941 | 365961 | 364143.3333 |  | SuN/C M-24h | 1.192892013 | 1.133986743 | 1.197410569 |
| SuN/C M-48h | 307844 | 295504 | 296712 | 300020 | 690148 | 674521 | 680174 | 681614.3333 |  | SuN/C M-48h | 2.241875755 | 2.28261208 | 2.292371053 |
| SuN/C M-72h | 313076 | 312176 | 323112 | 316121.3333 | 715562 | 755117 | 795242 | 755307 |  | SuN/C M-72h | 2.285585609 | 2.418882297 | 2.461196118 |
| SuN/C M-6h | 289966 | 287933 | 287893 | 288597.3333 | 743652 | 744001 | 743210 | 743621 |  | SuN/C M-96h | 2.564617921 | 2.58393793 | 2.581549395 |
| SuN/NPV M-6h | 275680 | 287654 | 236980 | 266771.3333 | 300168 | 290168 | 294101 | 294812.3333 |  | SuN/NPV M-6h | 1.088827626 | 1.008739666 | 1.241037218 |
| SuN/NPV M-12h | 290078 | 290168 | 292005 | 290750.3333 | 296360 | 285360 | 293324 | 291681.3333 |  | SuN/NPV M-12h | 1.021656244 | 0.983430289 | 1.004517046 |
| SuN/NPV M-24h | 291025 | 289874 | 286976 | 289291.6667 | 584351 | 585127 | 584635 | 584704.3333 |  | SuN/NPV M-24h | 2.007906537 | 2.018556338 | 2.03722611 |
| SuN/NPV M-48h | 312477 | 300781 | 301247 | 304835 | 760692 | 741115 | 720937 | 740914.6667 |  | SuN/NPV M-48h | 2.434393571 | 2.463968801 | 2.3931757 |
| SuN/NPV M-72h | 348521 | 349433 | 346952 | 348302 | 887814 | 898872 | 845623 | 877436.3333 |  | SuN/NPV M-72h | 2.547375911 | 2.572372958 | 2.437291037 |
| SuN/NPV M-96h | 307861 | 312007 | 312309 | 310725.6667 | 977408 | 998966 | 968971 | 981781.6667 |  | SuN/NPV M-96h | 3.174835396 | 3.201742269 | 3.102603511 |
